# Rheumatic Heart Disease Education Tools Integrated Into a Screening Program in Brazil: Acceptability and Knowledge Gain

**DOI:** 10.5334/gh.1510

**Published:** 2025-12-30

**Authors:** Jessica Abrams, Wanessa C. Vinhal, Craig A. Sable, Clareci S. Cardoso, Liesl Zühlke, Alison Spaziani, Laylah Ryklief, Maria Carmo P. Nunes, Isabely Karoline S. Ribeiro, Rebeca Previero, Lorena R. Silva, Luz M. Tacuri Chavez, Kaciane K. B. Oliveira, Ingred Beatriz Amaral, Larissa Lemos, Julliane S. Correa, Cecília T. Coelho, Brenno A. Santos, Luiza H. de Paula, Isadora S. Souza, Maria Luiza B. S. Santiago, Bruna C. Freitas, Gabriel R. Angelo, Matheus Henrique P. Nunes, Klicia J. Pereira, Antonio Luiz P. Ribeiro, Bruno R. Nascimento, On behalf of the PROVAR+ (Programa de RastreamentO da VAlvopatia Reumática and Other Cardiovascular Diseases) investigators

**Affiliations:** 1 Division of Paediatric Cardiology, Department of Paediatrics and Child Health, Faculty of Health Sciences, University of Cape Town, Cape Town, South Africa; 2 Reach, Cape Town, South Africa; 3 Universidade Federal de São João Del Rei, Campus Divinópolis, Divinópolis, MG, Brazil; 4 Congenital Heart Center, Ochsner Children’s Hospital, New Orleans, LA, United States; 5 South African Medical Research Council, Parow, Cape Town, South Africa; 6 Cardiology, Children’s National Health System, Washington, DC, United States; 7 Serviço de Cardiologia e Cirurgia Cardiovascular e Centro de Telessaúde do Hospital das Clínicas da UFMG, Belo Horizonte, MG, Brazil; 8 Departamento de Clínica Médica, Faculdade de Medicina da Universidade Federal de Minas Gerais, Belo Horizonte, MG, Brazil; 9 Curso de Medicina, Faculdade de Ciências Médicas de Minas Gerais, Belo Horizonte, MG, Brazil; 10 Curso de Medicina, Universidade Presidente Antônio Carlos, Barbacena, MG, Brazil; 11 Serviço de Hemodinâmica, Hospital Madre Teresa, Belo Horizonte, MG, Brazil

**Keywords:** Rheumatic Heart Disease, Education, Screening

## Abstract

**Background:**

Rheumatic heart disease (RHD) is a preventable cause of premature death among young individuals in low- and middle-income countries. Education is a key strategy to alleviate the burden of this disease. We aimed to assess the acceptability and knowledge gain of a series of low-literacy education flipcharts, presented during screening in high-burden areas of Brazil.

**Methods:**

Four low-literacy flipcharts were developed over three years and taught over 36 months to patients, community, school children, and health and education professionals, mostly in the state of Minas Gerais. In-person training and education workshops were assessed through printed surveys. Post-education surveys (for patients and community members), and post-training surveys (for healthcare and education professionals) were conducted from January 2023 to December 2025. A knowledge test, delivered at pre-training, post-training and three-month follow-up, was incorporated from January 2024 to March 2025.

**Results:**

Flipchart training was delivered to 1,317 healthcare and education professionals, while 1,292 patients and community members and 2,585 school students received education using the flipcharts. There was a statistically significant (p < 0.01) improvement in knowledge about rheumatic fever (RF) and RHD among healthcare and education professionals participating in the pre- and post-training survey (n = 511): RF as the cause of RHD (64% vs 95%), use of benzathine penicillin G (43% vs 98%), and frequency of antibiotic prophylaxis (21% vs 77%). The improvement from baseline was sustained at follow-up. Over the entire study period, 98% of survey respondents (2,134) reported learning something new, and 94% (2,041) intended to share the learnings with their peers or community.

**Conclusion:**

Culturally adapted, low-literacy educational flipcharts were successfully integrated into an existing RHD screening program in Brazil. The tool was well accepted among people living with RHD, their providers, and at-risk communities, with significant knowledge gain for healthcare and education professionals.

## Introduction

Chronic rheumatic heart disease (RHD) remains a major cause of cardiovascular disease in children and young adults, particularly in low- and middle-income countries. The 2021 Global Burden of Disease study estimated more than 54 million RHD cases and nearly 400,000 related deaths annually (1,2). Most of these cases occur in populations of resource-limited settings, with a high prevalence in sub-Saharan African and Latin America, including Brazil. These regional disparities reflect persistent inequities in environmental and social determinants of health, access to diagnostic services, and health literacy (3,4,5).

Lack of awareness and understanding about RHD among patients, families, and even healthcare providers remains a major barrier to optimal care (6,7). The critical role of education and an integrated prevention approach is reflected in the latest World Health Organization guideline on the prevention and diagnosis of rheumatic fever (RF) and RHD (8). Although various educational strategies targeting healthcare providers, patients, and families have been tested (9,10), there is little consensus on which approaches are most effective in improving knowledge, adherence and clinical outcomes.

To address this gap, Reach—a scientific and technical support hub (www.stoprhdwithreach.org]—developed a suite of educational tools on RF and RHD (**Supplementary Figure 1**) in collaboration with international partners. These tools, primarily flipcharts, are designed to support healthcare providers in communicating essential information about disease prevention, recognition, and management in underserved endemic settings. This study evaluated the acceptability of these educational tools, defined as ‘the extent to which people delivering or receiving a healthcare intervention consider it to be appropriate’ (11,12), and assesses the knowledge gained among patients, families and healthcare providers following their use in Brazil.

## Methods

This study was embedded within a pre-existing program, namely PROVAR+ (*Programa de Rastreamento da VAlvopatia Reumática e Outras Doenças Cardiovasculares;* Rheumatic Heart Disease Screening Program and Other Cardiovascular Diseases) (13). The PROVAR+ program operates in Belo Horizonte, Divinópolis (southeast), and Feira de Santana (northeast), Brazil. The educational component of the study was conducted over a 36-month period and integrated with ongoing screening and clinical activities in public schools, primary and secondary healthcare centers, and university hospital clinics.

### Educational material development

Four low-literacy flipcharts were developed by Reach (Geneva, Switzerland) in consultation with global experts on rheumatic fever and RHD as well as those with lived experience, over three years. The suite of flipcharts includes three patient-directed and one at-risk community directed flipchart, namely: (i) Introduction to RF and RHD, (ii) RHD and Pregnancy, (iii) RHD and Surgery, and (iv) RF and RHD Community Awareness (**Supplementary 1**). These flipcharts were subsequently adapted to the Brazilian context in collaboration with partners at the University Federal de Minas Gerais (UFMG).

### Facilitator Training

Personnel with a medical background working within PROVAR+ were designated as flipchart facilitators. They received bi-annual training on use of the flipcharts, structured workshops facilitations, and survey administration.

The structured flipchart sessions were aimed at training professionals who care for RHD patients on how to use the flipcharts, for dissemination to peer professionals through a train-the-trainer model; increasing awareness about RF/RHD among at-risk individuals; and improving patient adherence to prophylaxis and retention to care.

### Flipchart activities

All PROVAR+ screening activities were preceded by flipchart-based education for the consented attending community, including school children, patients, parents or guardians, siblings, and household members, as well as school staff.

Flipchart training for healthcare workers—physicians, nurses, nursing technicians, community health agents, and undergraduate health students—was conducted through once-off group workshops led by facilitators. In addition, educational activities for verbally consenting patients, families, and healthcare workers were carried out in the RHD outpatient clinics of the UFMG University Hospital (Hospital das Clínicas), a quaternary teaching institution and referral center for complex cardiovascular care.

In total, eleven primary care centers, three secondary care facilities, twelve schools, and one university hospital participated in this educational arm of the study. Sampling was by convenience with no age or gender, except for school children, who were included if aged 11 to 18 years.

### Evaluation

Patients, at-risk individuals, community members, and school children who underwent education with the appropriate flipchart were invited to complete the post-education survey (2023–2025, **Supplementary 2**). Healthcare workers and education professionals completed surveys post-training only (2023, **Supplementary 3**), and pre- and post-training (2024–2025, **Supplementary 4**). Participants who consented to follow-up were sent a system-generated link (RedCap® online software) three months from the date of initial training along with regular reminders to complete the survey. All surveys included questions relating to the acceptability and utility of the flipcharts. The pre-training, post-training and follow-up surveys (2024—2025) included a knowledge test of four questions assessing understanding of RHD etiology, preventative treatment, and prophylaxis frequency. Survey data were completed in a hard-copy format and entered into a RedCap® online database (14), except for the 3-month follow-up surveys which were completed electronically.

### Statistical analysis

Analysis was performed in the statistical software R-Studio® Version 2025.05.0 (Posit, Boston, MA, US). As an exploratory study without preliminary data from Brazil, no specific sample size calculation was applied, and the inclusion was made by convenience based on the screening and clinical schedule over three years. A descriptive analysis is presented for survey questions assessing the acceptability and utility of the new information post-flipchart education or training. For non-parametric data, a Wilcoxon signed rank test with a two-tailed significance level of 0.05 was performed to determine the differences in knowledge scores of healthcare workers and education professionals across two paired comparisons: (i) before and after the flipchart training, and (ii) before training and at three-month follow-up. In the case of partially completed knowledge questions, skipped questions were marked as zero. However, if an entire knowledge test was incomplete at any given time point, the participant was excluded from the relevant paired analysis.

## Results

From February 2023 to March 2025, flipchart training was delivered to 1,059 healthcare workers, 258 education professionals, 1,292 patients and community members, and 2,585 school students undergoing education. Table 1 presents the characteristics of survey respondents. Of the health and education professionals who underwent training on how to use the flipchart, 83% responded to the survey (N = 1,097).

**Table 1:** Characteristics of survey respondents following training or education using one or more flipchart and the cities in which they took place.


HEALTHCARE AND EDUCATION PROFESSIONALS (N = 1,097)	n (%)

Medical student	314 (29%)

Community health agent	155 (14%)

Nurse	146 (13%)

Nursing student	146 (13%)

School teacher	111 (10%)

Pharmacy student	53 (5%)

Nursing technician	46 (4%)

Unspecified	30 (3%)

General physician	29 (3%)

Specialist physician	19 (2%)

Dental student	19 (2%)

Allied healthcare professional	17 (2%)

Miscellaneous	7 (1%)

Biochemistry student	5 (0.5%)

**PEOPLE LIVING WITH OR AFFECTED BY RF/RHD, AND AT-RISK COMMUNITY (N = 1,244)**	

Outpatient	719 (56%)

School student	225 (18%)

Person living with RF/RHD	174 (14%)

Guardian	69 (5%)

Unspecified	50 (4%)

Hospital companion	7 (1%)

**CITY**	

Divinópolis	889 (38%)

Belo Horizonte	798 (34%)

Feira de Santana (Bahia)	452 (19%)

Other: Smaller cities	134 (6%)

Unspecified	68 (3%)


RF, rheumatic fever; RHD, rheumatic heart disease. Allied health professionals: Pathology technician, laboratory technician, psychologist, pharmacist, oral health assistant, speech therapist, nutritionist. Miscellaneous: Hospital administrator, art student, telecommunications, Master’s student, hospital employee.

**Figure 1:**
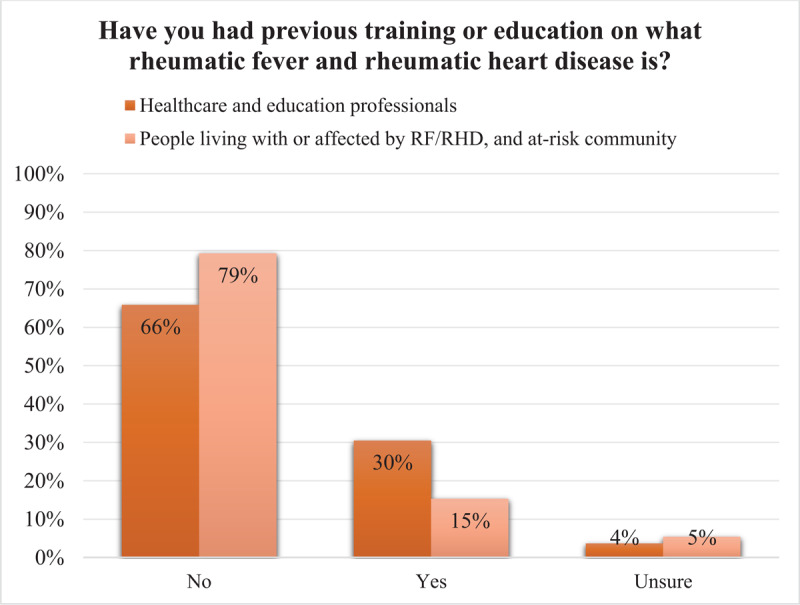
Survey response to the question of whether participants had any prior training or education about rheumatic fever (RF) or rheumatic heart disease (RHD).

Most respondents had no prior training or education about RF or RHD (Figure 1), with just 15% of people living with or affected by RF or RHD and at-risk community members reporting previous education about the disease.

### Acceptability

Table 2 presents survey data relating to acceptability of the RF/RHD flipcharts and self-reported learning of those undergoing flipchart training or education. Self-rated confidence (confident to very confident) in using the flipchart post-training was highest among healthcare students (76%) followed by qualified healthcare workers (74%). Almost all healthcare professionals (917; 96%) felt the flipcharts can help save lives.

**Table 2:** Post-training and post-education survey questions relating to acceptability of the RF/RHD flipcharts and self-reported learning, 2023–2025.


GROUP	CONFIDENT TO VERY CONFIDENT USING THE FLIPCHART, n(%)	NEW RHD LEARNING DUE TO TRAINING OR EDUCATION, n(%)	PLANNING TO SHARE LEARNINGS WITH PEERS, n(%)	BELIEVE THAT THE FLIPCHARTS CAN SAVE LIVES, n(%)

**Healthcare student (n = 527)**	399 (76%)	519 (98%)	520 (99%)	510 (97%)

**Community health agent (n = 155)**	64 (41%)	140 (90%)	144 (93%)	147 (95%)

**Qualified healthcare worker (n = 253)**	187 (74%)	248 (98%)	253 (100%)	245 (97%)

**Allied healthcare worker (n = 16)**	11 (69%)	16 (100%)	16 (100%)	15 (94%)

**Education professional (n = 111)**	47 (42%)	107 (96%)	107 (96%)	

**School student (n = 225)**		221 (98%)	146 (65%)	

**Outpatient (n = 719)**		710 (99%)	697 (97%)	

**Person living with RF/RHD (n = 174)**		173 (99%)	159 (91%)	

**Total**	**1,062 (67%)**	**2,134 (98%)**	**2,041 (94%)**	**917 (96%)**


RF, rheumatic fever; RHD, rheumatic heart disease. Healthcare students: medical, nursing, dental, pharmacy, and biochemistry; Qualified healthcare workers: general doctor, specialist doctor, dentist, nurse, nurse technician; Allied healthcare workers: Pathology technician, laboratory technician, psychologist, pharmacist, oral health assistant, speech therapist, nutritionist. Excluded from the table: Hospital administrator, art student, telecommunications, Master’s student, hospital employee, other (unspecified).

### Knowledge gain

The pre- and post-training survey was completed by 511 healthcare workers and education professionals from March 2024 to March 2025, of which 111 (mostly healthcare students) completed the follow-up survey at least three months after the initial training. Twenty-three individuals were excluded from the Wilcoxon signed rank test comparing paired pre- and post-training knowledge scores due to missing data. There was a statistically significant (p < 0.01) improvement in average knowledge score, increasing from 47% pre-training to 92% immediately post-training (Table 3). At the three-month follow-up, average scores declined to 79%, indicating some loss of retained knowledge. However, this follow-up score remained significantly higher than the pre-training score.

**Table 3:** Average knowledge scores before, immediately after, and three-months post flipchart training; A Wilcoxon signed rank test is presented, comparing knowledge scores at post-training and follow-up to pre-training.


TIMEPOINT	N	CORRECT ANSWERS (%):	CHANGE (%)*:	P-VALUE*:

**Pre-training**	488	47		

**Post-training**	488	92	45	<0.01

**Follow-up**	111	79	32	<0.01


*Compared to pre-training.

**Figure 2:**
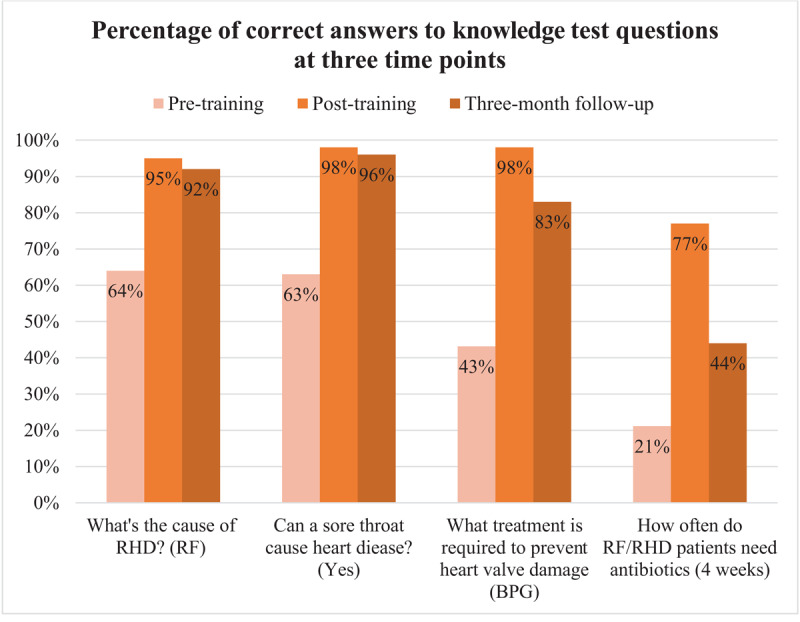
Healthcare worker and education professionals correct response to knowledge test questions pre-training, post-training (n = 511), and three or more months following initial training (n = 111). Correct answers are indicated in brackets. RF, rheumatic fever; RHD, rheumatic heart disease; BPG, benzathine penicillin G.

Knowledge per test question is illustrated in Figure 2. The greatest improvement in knowledge was observed in identifying the appropriate type and frequency of treatment required to prevent and control RF/RHD. Before training, 43% of respondents correctly reported benzathine penicillin G (BPG) as the recommended treatment to prevent heart valve damage; this rose to 98% post-training. Similarly, regarding frequency of administration of BPG to RF/RHD patients, 21% correctly answered ‘every 4 weeks’, which improved to 77% post- training.

### Knowledge retention

Knowledge retention three or more months post-training was high for all questions except duration of therapy (77% to 44%).

## Discussion

The flipcharts were well accepted among healthcare workers, educators, patients, and community members, as demonstrated by the willingness to share the tool with peers, patients, and families, and their potential benefit to people living with RHD was recognized. Notably, our pre-post results showed a statistically significant improvement in knowledge about the cause, prevention, and management of RHD. Together, these findings suggest that this approach may benefit people living with RHD and their providers in other endemic countries, but measurement of health outcomes are required.

The Reach educational flipcharts are designed to facilitate the interaction between the provider and their patient and caregivers, explaining RF/RHD in an easy-to-understand manner. Each flipchart targets a specific group—people living with RF/RHD, patients who are pregnant, or those undergoing heart surgery—covering pathophysiology and risk factors, the importance of treatment and follow-up care, and information about the surgery types and recovery period. During early pilot testing in Brazil, it became apparent that a general, community awareness flipchart was needed. Additionally, it became apparent that providers who were trained on use of the flipcharts appeared to benefit from the content, which prompted further investigation (15).

The pre- and post-training surveys completed by healthcare and education professionals identified gaps in knowledge about RF/RHD, with less than half of participants identifying the type (43%) and duration (21%) of secondary prophylaxis. Additionally, respondents displayed only moderate knowledge that a sore throat can lead to RHD (63%). These baseline findings align with studies investigating healthcare worker knowledge, attitudes, practice and education needs in high-burden settings. A hospital-based cross-sectional study in Nigeria reported inadequate knowledge in two-thirds of healthcare workers, with only 27% correctly identifying Strep A as the causative agent for RHD (16). Similarly, frontline physicians at an Egyptian teaching hospital scored low in knowledge about antibiotic duration (29.6%) (17). A multicenter survey assessing RHD service readiness across four districts in Uganda noted limited knowledge of RHD among healthcare workers as a major contributor to delayed identification and treatment, calling for pre-service and in-service training (18). This is echoed in findings among nurses in Ethiopian cardiac centers, where over half (48.7%) scored below the mean on nurse-level knowledge questions (19).

It is common to see poor retention in knowledge following education or training interventions (10,20). In contrast, our findings show key information was sustained after three months. Several factors, or a combination thereof, may explain this retention: the flipcharts were culturally adapted, designed for low-literacy audiences, and remained with healthcare providers as a lasting reference after training. Flipchart-based education has previously been incorporated into other healthcare worker training or at-risk community and patient-facing interventions, but has not been evaluated as a standalone activity (21,22,23). Outside of RHD, a randomized controlled trial in India found that mothers who received flipchart-based antenatal education had significant improvements in essential newborn care knowledge and practices, with these gains sustained after six months (24).

Despite the known burden of RHD in Brazil and historical RHD school-based education activities in the region (10,25,26), at baseline, just 30% of healthcare and education professionals had any previous formal training or education on RF/RHD. This was 15% among people living with or affected by RF/RHD and at-risk community. At least some of the information presented via the flipcharts was new to 98% of all participants. Together, these findings suggest the flipcharts can provide basic but crucial RHD-related information applicable at all levels.

### Limitations

Low uptake of the follow-up survey is noted, possibly attributable to challenges in maintaining contact with participants across the several locations where the flipcharts were applied, lack of time or interest in responding, and potential concerns about anonymity. Differences in professional background and baseline knowledge may have also influenced participation and retention. Nevertheless, among those who completed the three-month follow-up survey, knowledge retention remained evident, reinforcing the effectiveness of the educational intervention. Second, no stratified sampling procedures were performed, limiting the extrapolation of our findings to the entire Brazilian underserved population or to other settings. Third, the study design limited the outcomes of interest to acceptability and knowledge gain, not allowing for broader inferences about impact on RHD care or health outcomes. Thus, further assessments are warranted, with special focus on retention of care, adherence to prophylaxis, and prescription of guideline-driven therapy.

### Implications for practice

This is the largest RHD educational program in Latin America with a train-the-trainer design. The willingness of nearly all respondents (94%) to share their learnings indicates a strong potential for secondary dissemination, suggesting this model could extend its impact beyond directly educated or trained participants. Within Brazil’s Family Health Program structure (part of the universal Unified Health System, *Sistema Único de Saúde*) (27), which emphasizes community outreach, the train-the-trainer model could be a scalable mechanism to embed RHD education into routine public health practice. As for other diseases with social determinants, global joint efforts for the development of such strategies are essential, combining lessons learned from different regional programs. Sharing experiences with different groups and organizations, with the technical support counselling of experts with diverse backgrounds, might facilitate the optimization of processes and the design of the ideal tools for different endemic settings.

## Conclusion

This study demonstrates that culturally adapted, low-literacy educational tools integrated into existing RHD screening programs are feasible and well accepted in high-risk Brazilian populations. The intervention led to measurable gains in RHD-related knowledge among health and education professionals and shows promise for broader application through train-the-trainer dissemination models. Further investigations into long-term knowledge retention and impact on patient health outcomes are required.

## Additional File

The additional file for this article can be found as follows:

10.5334/gh.1510.s1 Supplementary Files. Supplementary 1 to 4.
