# Supplementary material for: Rheumatic Heart Disease Education Tools Integrated Into a Screening Program in Brazil: Acceptability and Knowledge Gain
Source: Glob Heart. 2025 Dec 30;20(1):115. doi: 10.5334/gh.1510 (PMC12758106; doi:10.5334/gh.1510)
Supplement: Supplementary Files. — Supplementary 1 to 4. [file gh-20-1-1510-s1.pdf]

Supplementary 1

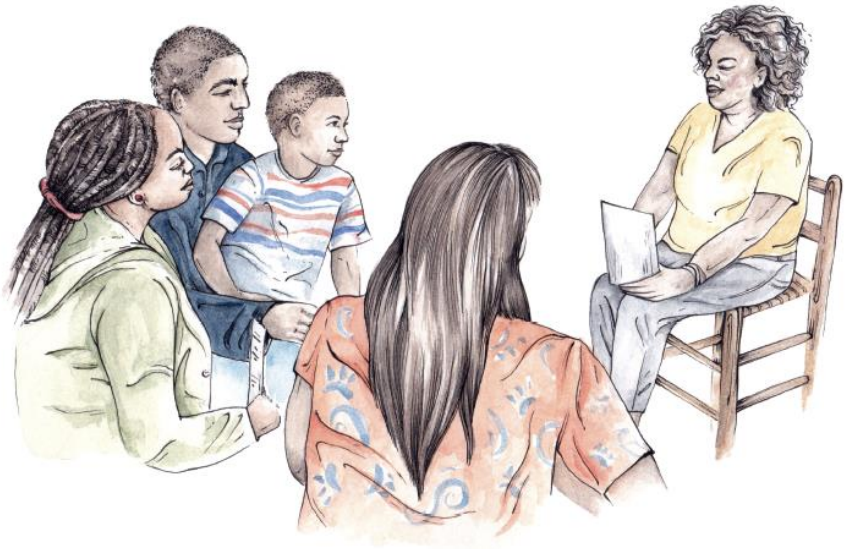

reach.
To stop rheumatic heart disease

Rheumatic Fever and Rheumatic Heart Disease

Community Awareness Flipchart

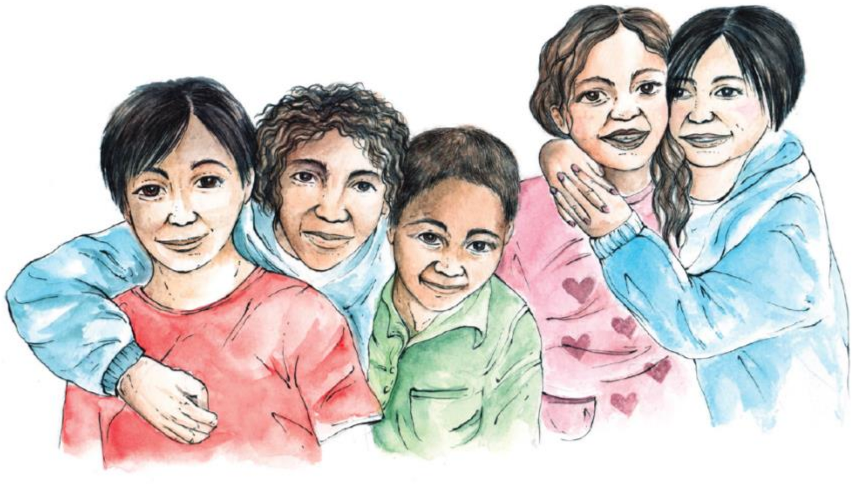

reach.
To stop rheumatic heart disease

Rheumatic Fever and Rheumatic Heart Disease

Patient Education Flipchart

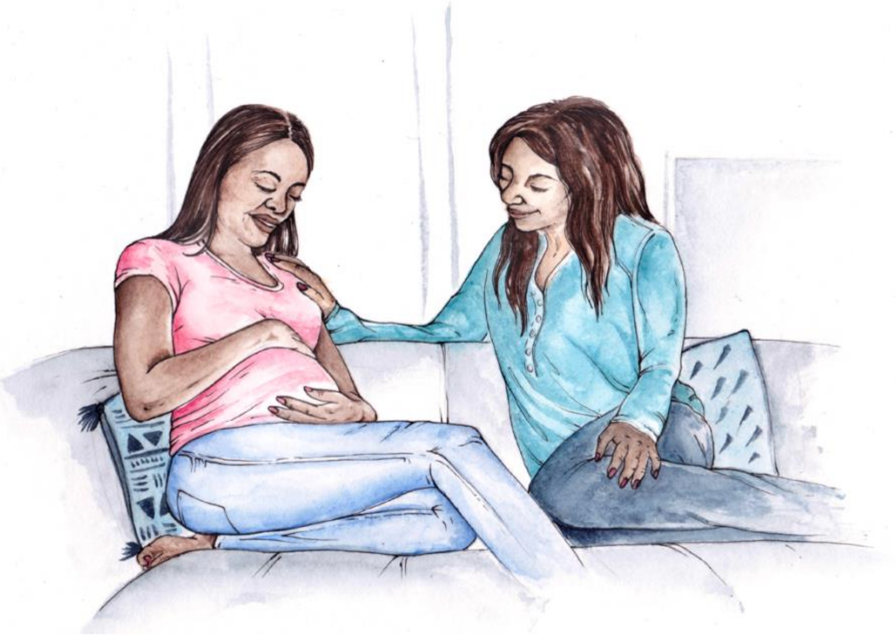

reach .

To stop rheumatic heart disease

Rheumatic Heart Disease and Pregnancy

A patient education flipchart for pregnant women with RHD

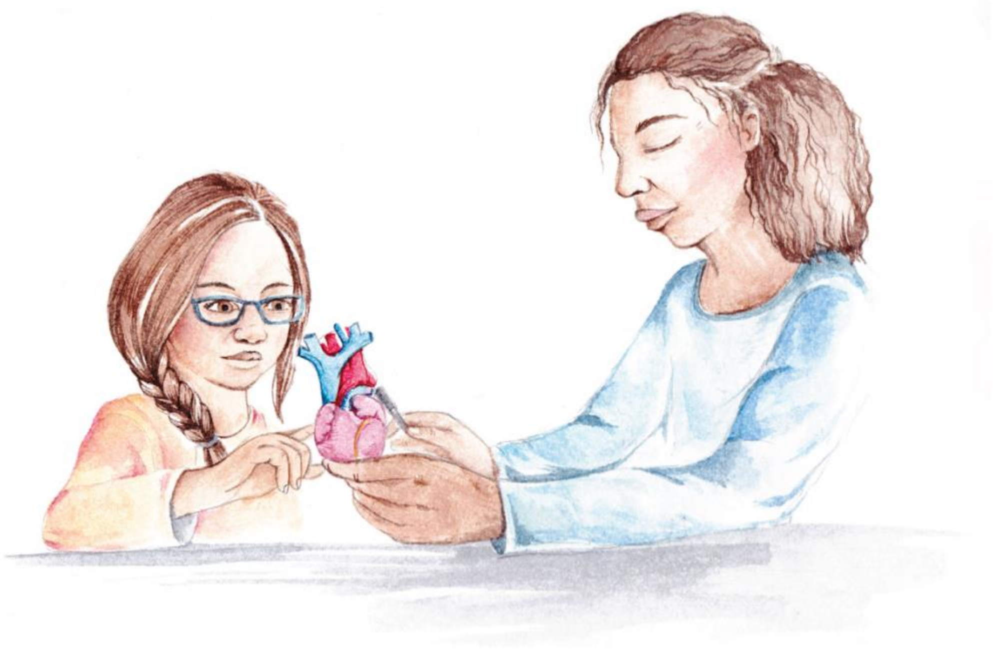

reach .

To stop rheumatic heart disease

Rheumatic Heart Disease and Surgery

Patient Education Flipchart

Rheumatic Fever/Rheumatic Heart Disease Flipchart Survey for Patients and Community

Obrigado por ouvir esta educação sobre febre reumática e doença cardíaca reumática. Por favor, responda a estas perguntas curtas.

Thank you for listening to this education about rheumatic fever and rheumatic heart disease. Please would you complete these short questions.

Em que data você concluiu o treinamento? / What date did you complete the training?

____

Eu sou: / (I am a):

- ◯ Estudante de escola/ School student
- ◯ Pessoa vivendo com febre reumática / Cardiopatia Reumática / Person living with RF/RHD
- ◯ Paciente externo / Outpatient
- ◯ Outro (especificar) / Other (specify)

Outro (especificar) / Other (specify)

____

Você teve educação prévia sobre o que a febre reumática aguda e cardiopatia reumática são? / Have you had previous education on what rheumatic fever and rheumatic heart disease is?

- ◯ Não / No
- ◯ Sim / Yes
- ◯ Não sei ao certo / Unsure

Cidade onde você está participando deste processo educativo (ferramenta educativa com brochuras)? / City in which you are participating in this (flipchart education tool) education?

- ◯ Belo Horizonte
- ◯ Divinópolis
- ◯ Outra (especificar) / Other (specify)

Outro (especificar) / Other (specify)

____

Por favor, verifique qual brochura educacional lhe foi mostrada: / Please tick which education flipchart(s) you have been shown:

- ☐ Febre Reumática e Cardiopatia Reumática (sim/não) / Rheumatic Fever and Rheumatic Heart Disease
- ☐ Cardiopatia Reumática e Gravidez (sim/não) / Rheumatic Heart Disease and Pregnancy
- ☐ Cardiopatia Reumática e Cirurgia (sim/não) / Rheumatic Heart Disease and Surgery
- ☐ Brochura de Conscientização para a Comunidade (sim/não) / Community Awareness Flipchart

---

Este educação melhorou sua compreensão sobre a Cardiopatia Reumática? / Did this education help your understanding of Rheumatic Heart Disease?

- ◯ Não, eu já sabia muito sobre a doença / No, I already knew a lot about the disease
- ◯ Sim, eu aprendi novas informações / Yes, I learned some new information
- ◯ Sim, todas as informações foram novas para mim / Yes, All the information was new to me

---

Com base nas informações educacionais, você discutirá esta doença com sua família, amigos ou comunidade? / Based on the education information, will you discuss this disease with your family, friends, or community?

- ◯ Não / No
- ◯ Sim / Yes
- ◯ Não tenho Certeza / Unsure

Você tem algum feedback ou comentário adicional sobre a brochura ou o educação? / Do you have any additional feedback or comments on the flipchart or education?

____

Rheumatic Fever/Rheumatic Heart Disease Flipchart Post-Training Survey for Health and Education Professionals

Obrigado por tomar seu tempo para participar das sessões de treinamento. Esperamos que elas ajudem as vidas de pessoas impactadas pela febre reumática aguda e pela cardiopatia reumática. Você poderia, por favor, responder a estas breves questões após a conclusão do treinamento?

Thank you for taking time to attend the training sessions, we hope that this will help the lives of people impacted by rheumatic fever and rheumatic heart disease. Please would you complete these short questions as a closing to the training.

Em que data você concluiu o treinamento? / What date did you complete the training?

____

Eu sou: / (I am a):

- ◯ Médico (generalista) / Doctor (general)
- ◯ Médico (especialista) / Doctor (specialist)
- ◯ Agente de Saúde / Health agent
- ◯ Estudante de Medicina / Medical student
- ◯ Enfermeiro(a) / Nurse
- ◯ Estudante de enfermagem / Nursing student
- ◯ Professor ou representante da escola / Teacher or school representative
- ◯ Outro (especificar) / Other (specify)

Outro (especificar) / Other (specify)

____

Você teve treinamento prévio sobre o que a febre reumática aguda e cardiopatia reumática são? / Have you had previous training on what rheumatic fever and rheumatic heart disease is?

- ◯ Não / No
- ◯ Sim / Yes
- ◯ Não sei ao certo / Unsure

Cidade onde você está participando deste treinamento (ferramenta educativa com brochuras)? / City in which you are participating in this (flipchart education tool) training?

- ◯ Belo Horizonte
- ◯ Divinópolis
- ◯ Outra (especificar) / Other (specify)

Outra (especificar) / Other (specify)

____

Local desta sessão de treinamento: / Site or location of this training session:

- ◯ Hospital / Hospital
- ◯ Clínica / Clinic
- ◯ Universidade / University
- ◯ Escola / School
- ◯ Outro (especificar) / Other (specify)

Outro (especificar) / Other (specify)

____

Por favor marque em qual brochura educacional você foi treinado (a): / Please tick which education flipchart(s) you have been trained on:	☐ Febre Reumática e Cardiopatia Reumática (sim/não) / Rheumatic Fever and Rheumatic Heart Disease<br>☐ Cardiopatia Reumática e Gravidez (sim/não) / Rheumatic Heart Disease and Pregnancy<br>☐ Cardiopatia Reumática e Cirurgia (sim/não) / Rheumatic Heart Disease and Surgery<br>☐ Brochura de Conscientização para a Comunidade (sim/não) / Community Awareness Flipchart
Você foi treinado com alguma destas ferramentas educacionais no ultimo ano? / Were you trained on any of these educational tools last year?	◯ Não / No<br>◯ Sim / Yes
Este treinamento melhorou sua compreensão sobre a Cardiopatia Reumática? / Did this training help your understanding of Rheumatic Heart Disease?	◯ Não, eu já sabia muito sobre a doença / No, I already knew a lot about the disease<br>◯ Sim, eu aprendi novas informações / Yes, I learned some new information<br>◯ Sim, todas as informações foram novas para mim / Yes, All the information was new to me
Você acredita que estas ferramentas educacionais podem melhorar as vidas dos pacientes? / Do you think these educational tools can help improve patients' lives?	◯ Não / No<br>◯ Sim / Yes<br>◯ Não tenho certeza / Unsure
Quão confiante ou confortável você se sente ao usar a ferramenta educacional com alguém que não tem conhecimentos sobre febre reumática e cardiopatia reumática? / How confident or comfortable do you feel using the educational tool with someone who does not know about rheumatic fever and rheumatic heart disease?	◯ muito desconfortável/muito inseguro / very uncomfortable / very unconfident<br>◯ desconfortável/não confiante / uncomfortable / not confident<br>◯ um pouco confortável/confiante / somewhat comfortable / confident<br>◯ confortável/confiante / comfortable / confident<br>◯ muito confortável/confiante / very comfortable / confident
Qual a probabilidade de você compartilhar seu treinamento na ferramenta educacional com seus colegas? / How likely are you to share your training on the educational tool with your colleagues?	◯ Não pretendo compartilhar o treinamento com meus colegas / I do not plan to share the training with my colleagues<br>◯ Posso compartilhar o treinamento com meus colegas / I might share the training with my colleagues<br>◯ Com certeza compartilharei o treinamento com meus colegas / I will definitely share the training with my colleagues
Quantos pacientes com febre reumática ou cardiopatia reumática você estima atender em um mês? (pular se não aplicável) Texto (número) / How many patients with rheumatic fever or rheumatic heart disease do you estimate you will see in one month? (skip if not applicable) Text (number)	______________________________
Se você for um professor ou representante da escola, compartilhará esta brochura com seus alunos? (pular se não aplicável) / If you are a teacher or school representative, will you share this flipchart with your students? (skip if not applicable)	◯ Não / No<br>◯ Sim / Yes<br>◯ Não tenho Certeza / Unsure

Quantos alunos da escola você atende por mês? (pular se não aplicável) Texto (número) / How many school students do you see per month? (skip if not applicable) Text (number)	__________________________________
Você tem algum feedback ou comentário adicional sobre a brochura ou o treinamento? / Do you have any additional feedback or comments on the flipchart or training?	__________________________________

RF/RHD Flipchart Post-Training Survey for Health and Education Professionals - INITIAL (PRE-TRAINING) SURVEY

Please complete the survey below.

Thank you!

DATA DO TREINAMENTO / DATE OF TRAINING: ______________________

TREINADOR LÍDER / LEAD TRAINER: ______________________

CIDADE / CITY

- ◯ Belo Horizonte
- ◯ Divinopolis
- ◯ Bahia
- ◯ Outro (especificar):

Outro (especificar): ______________________

LOCAL / VENUE

- ◯ Hospital / Hospital
- ◯ Clínica / Clinic
- ◯ Universidade / University
- ◯ Escola / School
- ◯ Centro Comunitário / Community center
- ◯ Outro (especificar):

Outro (especificar): ______________________

Eu sou: / (I am a):

- ◯ Médico (generalista) / Doctor (general)
- ◯ Médico (especialista) / Doctor (specialist)
- ◯ Agente de Saúde / Health agent
- ◯ Estudante de Medicina / Medical student
- ◯ Enfermeiro(a) / Nurse
- ◯ Estudante de enfermagem / Nursing student
- ◯ Fisioterapeuta / Physiotherapist
- ◯ Dentista / Dentist
- ◯ Professor ou representante da escola / School teacher or representative
- ◯ Outro (especificar) / Other (specify)

Outro (especificar) / Other (specify): ______________________

---

2. Área de abrangência (cidade/região em que você trabalha) / Catchment area (city/region you work in): ______________________

Você teve treinamento prévio sobre o que a febre reumática aguda e cardiopatia reumática são? / Have you had previous training about rheumatic fever and rheumatic heart disease?

- ◯ Sim / Yes
- ◯ Não / No
- ◯ Não sei ao certo / Unsure

Qual é a causa da Cardiopatia Reumática? / What is the cause of rheumatic heart disease?	◯ Pessoas já nascem com a doença / people are born with the disease<br>◯ Idade / age<br>◯ Resposta autoimune (Febre Reumática aguda) / autoimmune response (acute rheumatic fever)<br>◯ Não sei / Unsure
Dor de garganta (Faringoamigdalite causada pelo estreptococo) pode causar doenças cardíacas? / Can a sore throat (strep throat) cause heart disease?	◯ Sim / Yes<br>◯ Não / No<br>◯ Não sei / Unsure
Qual tratamento é necessário para prevenir danos às valvas cardíacas? / What treatment is needed to prevent heart valve damage?	◯ Penicilina benzatina ou benzetacil / benzathine penicillin G<br>◯ Ibuprofeno / ibuprophen<br>◯ Vancomicina / vancomycin<br>◯ Não sei / unsure
Com qual frequência o paciente com Febre Reumática aguda ou Cardiopatia Reumática deve tomar antibióticos? / How often do patient with acute rheumatic fever or rheumatic heart disease have to take antibiotics?	◯ A cada 2 semanas / Every 2 weeks<br>◯ A cada 4 semanas / Every 4 weeks<br>◯ A cada 3 meses / Every 3 months<br>◯ Não sei / Unsure

RF/RHD Flipchart Post-training Survey For Health And Educational Professional - POST (POST-TRAINING)

Please complete the survey below.

Thank you!

Eu sou: / (I am a):	◯ Médico (generalista) / Doctor (general)<br>◯ Médico (especialista) / Doctor (specialist)<br>◯ Agente de Saúde / Health agent<br>◯ Estudante de Medicina / Medical student<br>◯ Enfermeiro(a) / Nurse<br>◯ Estudante de enfermagem / Nursing student<br>◯ Fisioterapeuta / Physiotherapist<br>◯ Dentista / Dentist<br>◯ Professor ou representante da escola / School teacher or representative<br>◯ Outro (especificar) / Other (specify)
Outro (especificar) / Other (specify):	__________________________________
Por favor marque em qual brochura educacional você foi treinado (a): / Please tick which education flipchart(s) you have been trained on:	☐ Febre Reumática e Cardiopatia Reumática / Rheumatic Fever and Rheumatic Heart Disease<br>☐ Cardiopatia Reumática e Gravidez / Rheumatic Heart Disease and Pregnancy<br>☐ Cardiopatia Reumática e Cirurgia / Rheumatic Heart Disease and Surgery<br>☐ Brochura de Conscientização para a Comunidade / Community Awareness Flipchart
Você foi treinado com alguma destas ferramentas educacionais no ultimo ano? / Have you been previously trained on any of these educational tools in the last year?	◯ Sim / Yes<br>◯ Não / No
Este treinamento melhorou sua compreensão sobre a Cardiopatia Reumática? / Did this training help your understanding of RHD?	◯ Não, eu já sabia muito sobre a doença / No, I already knew a lot about the disease<br>◯ Sim, eu aprendi novas informações / Yes, I learned some new information<br>◯ Sim, todas as informações foram novas para mim / Yes, All the information was new to me
Qual é a causa da Cardiopatia Reumática? / What is the cause of rheumatic heart disease?	◯ Pessoas já nascem com a doença / people are born with the disease<br>◯ Idade / age<br>◯ Resposta autoimune (Febre Reumática aguda) / autoimmune response (acute rheumatic fever)<br>◯ Não sei / Unsure
Dor de garganta (Faringoamigdalite causada pelo estreptococo) pode causar doenças cardíacas? / Can a sore throat (strep throat) cause heart disease?	◯ Sim / Yes<br>◯ Não / No<br>◯ Não sei / Unsure
Qual tratamento é necessário para prevenir danos às valvas cardíacas? / What treatment is needed to prevent heart valve damage?	◯ Penicilina benzatina ou benzetacil / benzathine penicillin G<br>◯ Ibuprofeno / ibuprophen<br>◯ Vancomicina / vancomycin<br>◯ Não sei / unsure

Com qual frequência o paciente com Febre Reumática aguda ou Cardiopatia Reumática deve tomar antibióticos? / How often do patient with acute rheumatic fever or rheumatic heart disease have to take antibiotics?	◯ A cada 2 semanas / Every 2 weeks<br>◯ A cada 4 semanas / Every 4 weeks<br>◯ A cada 3 meses / Every 3 months<br>◯ Não sei / Unsure
Você acredita que estas ferramentas educacionais podem melhorar as vidas dos pacientes? / Do you think these educational tools can help improve patients' lives?	◯ Sim / Yes<br>◯ Não / No<br>◯ Não tenho certeza / Unsure
Quão confiante ou confortável você se sente ao usar a ferramenta educacional com alguém que não tem conhecimentos sobre febre reumática e cardiopatia reumática? / How confident or comfortable do you feel using the educational tool with someone who does not know about rheumatic fever and rheumatic heart disease?	◯ muito desconfortável/muito inseguro / very uncomfortable / very unconfident<br>◯ desconfortável/não confiante / uncomfortable / not confident<br>◯ um pouco confortável/confiante / somewhat comfortable / confident<br>◯ confortável/confiante / comfortable / confident<br>◯ muito confortável/confiante / very comfortable / confident
Qual a probabilidade de você compartilhar seu treinamento na ferramenta educacional com seus colegas? / How likely are you to share your training on the educational tool with your colleagues?	◯ Não pretendo compartilhar o treinamento com meus colegas / I do not plan to share the training with my colleagues<br>◯ Posso compartilhar o treinamento com meus colegas / I might share the training with my colleagues<br>◯ Com certeza compartilharei o treinamento com meus colegas / I will definitely share the training with my colleagues
Você tem algum feedback ou comentário adicional sobre a brochura ou o treinamento? / Do you have any additional feedback or comments on the flipchart or training?	______________________________
Podemos entrar em contato com você para obter informações de acompanhamento? / May we contact you for follow-up information?	◯ Sim / Yes<br>◯ Não / No
Mensagem de texto (número de telefone) / Text message (phone number)	______________________________
Endereço de email / Email address	______________________________

RF/RHD Flipchart Post-Training Survey for Health and Education Professionals - FOLLOW-UP SURVEY (Approx. 3-Months)

Please complete the survey below.

Thank you!

Eu sou: / (I am a):	◯ Médico (generalista) / Doctor (general)<br>◯ Médico (especialista) / Doctor (specialist)<br>◯ Agente de Saúde / Health agent<br>◯ Estudante de Medicina / Medical student<br>◯ Enfermeiro(a) / Nurse<br>◯ Estudante de enfermagem / Nursing student<br>◯ Fisioterapeuta / Physiotherapist<br>◯ Dentista / Dentist<br>◯ Professor ou representante da escola / School teacher or representative<br>◯ Outro (especificar) / Other (specify)
Outro (especificar) / Other (specify)	__________________________________
Initial training date:<br><br>[training_date]	
Data de hoje / Today's date:	__________________________________
Local do treinamento inicial / Location of initial training:	◯ Belo Horizonte<br>◯ Divinopolis<br>◯ Bahia<br>◯ Outro
Outro (especificar):	__________________________________
Módulo(s) em que você foi treinado: / Module(s) you trained on:	☐ Febre Reumática e Cardiopatia Reumática / Rheumatic Fever and Rheumatic Heart Disease<br>☐ Cardiopatia Reumática e Gravidez / Rheumatic Heart Disease and Pregnancy<br>☐ Cardiopatia Reumática e Cirurgia / Rheumatic Heart Disease and Surgery<br>☐ Brochura de Conscientização para a Comunidade / Community Awareness Flipchart
Qual é a causa da Cardiopatia Reumática? / What is the cause of rheumatic heart disease?	◯ Pessoas já nascem com a doença / people are born with the disease<br>◯ Idade / age<br>◯ Resposta autoimune (Febre Reumática aguda) / autoimmune response (acute rheumatic fever)<br>◯ Não sei / Unsure
Dor de garganta (Faringoamigdalite causada pelo estreptococo) pode causar doenças cardíacas? / Can a sore throat (strep throat) cause heart disease?	◯ Sim / Yes<br>◯ Não / No<br>◯ Não sei / Unsure

Qual tratamento é necessário para prevenir danos às valvas cardíacas? / What treatment is needed to prevent heart valve damage?	◯ Penicilina benzatina ou benzetacil / benzathine penicillin G<br>◯ Ibuprofeno / ibuprophen<br>◯ Vancomicina / vancomycin<br>◯ Não sei / unsure
Com qual frequência o paciente com Febre Reumática aguda ou Cardiopatia Reumática deve tomar antibióticos? / How often do patient with acute rheumatic fever or rheumatic heart disease have to take antibiotics?	◯ A cada 2 semanas / Every 2 weeks<br>◯ A cada 4 semanas / Every 4 weeks<br>◯ A cada 3 meses / Every 3 months<br>◯ Não sei / Unsure
Desde a sua formação, você utilizou os flipcharts? / Since your training, have you used the flipcharts?	◯ Sim / Yes<br>◯ Não / no

Com que frequência você usa as seguintes ferramentas: / How often do you use the following tools:

	Nunca / Never	Ocasionalmente / Occasionally	Uma vez por semana / Once per week	Duas vezes por semana / Twice per week	Diariamente / Every day
Febre Reumática e Cardiopatia Reumática	☐	☐	☐	☐	☐
Cardiopatia Reumática e Gravidez	☐	☐	☐	☐	☐
Cardiopatia Reumática e Cirurgia	☐	☐	☐	☐	☐
Brochura de Conscientização para a Comunidade	☐	☐	☐	☐	☐

12. Número aproximado de pessoas com quem compartilhei esta ferramenta desde meu treinamento: / Approximate number of people I have shared this tool since my training:

	0	1-10	11-20	21-30	31+
Febre Reumática e Cardiopatia Reumática	☐	☐	☐	☐	☐
Cardiopatia Reumática e Gravidez	☐	☐	☐	☐	☐
Cardiopatia Reumática e Cirurgia	☐	☐	☐	☐	☐
Brochura de Conscientização para a Comunidade	☐	☐	☐	☐	☐

Você tem alguma necessidade adicional de ferramentas educacionais? / Do you have any additional needs for educational tools? ______________________________
